# Prognostic Value of Galectin-3 in Patients with Heart Failure

**DOI:** 10.1155/2015/690205

**Published:** 2015-04-16

**Authors:** Ivica Bošnjak, Kristina Selthofer-Relatić, Aleksandar Včev

**Affiliations:** ^1^Department for Cardiovascular Disease, Clinic for Internal Medicine, University Hospital Centre Osijek, J. Huttlera 4, 31000 Osijek, Croatia; ^2^Faculty of Medicine Osijek, University Josipi Juraj Strossmayer Osijek, J. Huttlera 4, 31000 Osijek, Croatia; ^3^Department for Gastroenterology, Clinic for Internal Medicine, University Hospital Centre Osijek, Osijek, Croatia

## Abstract

Galectins are a family of soluble *β*-galactoside-binding lectins that have important role in inflammation, immunity, and cancer. Galectin-3 as a part of this lectin family plays a very important role in development of heart failure. According to recent papers, galectin-3 plasma level correlates with heart failure outcome, primarily with rehospitalisation and death from heart failure. This paper summarizes the most recent advances in galectin-3 research, with the accent on the role of galectin-3 in pathophysiology of myocardial remodelling and heart failure development—with preserved and reduced ejection fraction, and some implication on development of new disease modifying drugs.

## 1. Introduction

Heart failure (HF) can be defined as a complex mechanical and neurohumoral syndrome manifested by hemodynamic congestion presenting with dyspnea, orthopnea, paroxysmal nocturnal dyspnea, or peripheral oedema, along with the presence of objective evidence of cardiac dysfunction [1, 2]. A new symptom in heart failure, bendopnea, has been described recently. Bendopnea is mediated via a further increase in filling pressures during bending when filling pressures are already high, particularly if cardiac index is reduced [3]. Heart failure remains one of the most prevalent and challenging medical conditions. Despite advances in treatment, morbidity and mortality in HF are very high, thus representing one of the most costly medical conditions [4]. Until now, HF has been considered to be associated with impaired cardiac contractility and cardiac dilation, but it has become evident that a considerable portion of patients presenting with clinical HF have a normal ejection fraction (EF). Now we can distinguish heart failure with reduced ejection fraction < 40% (HFrEF) and heart failure with preserved ejection fraction > 40% (HFpEF) [5, 6]. The pathophysiology concept of HF is related to the concept of myocardial remodelling. Increased stress or injury to the myocardium due to hypertension, diabetes mellitus, or ongoing ischemia can contribute to cardiac remodelling [7]. Response to acute or chronic damage can involve activation of immune cells to the myocardium, production of cell signaling proteins from local pericytes, mast cells, and macrophages, resulting in activation of resident fibroblasts and myofibroblasts, and deposition of collagen into extracellular matrix, which is correlated with collagen generating cardiac fibrosis [8]. There are many regulators involved in the pathophysiology of cardiac fibrosis. One of them is galectin-3.

## 2. Galectin-3 and Myocardial Remodelling

Galectin-3 (Gal-3) is a member of a galectin family involved in numerous physiological and pathological processes, some of which, inflammation and fibrosis, are pivotal contributing pathophysiological mechanisms to the development of HF. Gal-3 is a 29–35 kDa chimaera-type galectin which is unique in that it is the only member of the galectin family with an extended N-terminal domain constituted of tandem repeats of short amino acid segments linked to a single C-terminal carbohydrate-recognition domain. C-terminal domain is responsible for lectin activity, while the presence of the N-terminal domain is necessary for full biological activity of galectin-3 [9, 10]. It is found in a wide range of tissues [11]. Gal-3 lacks a secretion signal peptide for classical vesicle-mediated exocytosis, so it is localized primary not only in the cytoplasm but also in the nucleus of mitochondria. When secreted in the extracellular space Gal-3 can interact with cell surface receptors to initiate transmembrane signalling pathways for different cellular functions. Gal-3 is also necessary for normal macrophage function [12]. In granulocyte-macrophage colony-stimulating factor transgenic mice, the expression of Gal-3 was increased by 6-fold following the activation of macrophages [13]. Significant infiltration of macrophages was observed in hypertrophied heart, as well as in active myocarditis, and colocalized Gal-3 was found [1, 14]. Consequently, it can be concluded that Gal-3 has dependent stimulatory effect on macrophage migration. This hypothesis was confirmed by Sharma et al. by showing that exogenous recombinant Gal-3 significantly increased macrophage migration, an effect that was inhibited by the endogenous peptide N-acetyl-seryl-aspartyl-lysyl-proline (Ac-SDKP) [15]. Henderson et al. found that macrophages had abundant Gal-3 within their nucleus and cytoplasm and that they were able to secrete substantial amounts of Gal-3 into the supernatant in the cell culture suggesting a location in the extracellular space [16]. In a murine model of hepatic fibrosis, Gal-3 was found to be localized by proliferating fibroblasts [17]. In normal rat cardiac fibroblasts, addition of exogenous recombinant Gal-3 significantly increased fibroblast proliferation, which resulted in an increased collagen production in hypertrophied heart [15]. Simultaneously, Gal-3 binding sites were localized around the nucleus of proliferating fibroblast, whereas resting cells only had minimal binding sites in the cytoplasm [18]. It was suggested that Gal-3 induced cardiac fibrosis via activation of cyclin D1, thus allowing a macrophage derived mediator to affect the myocardium [18]. Similarly, involvement of Gal-3 in development of fibrosis has been demonstrated not only in the heart [18] but also in the liver and in the kidney [16, 17].

Cardiac remodeling is a crucial element in the clinical outcome of heart failure, and therefore various drugs are administered to prevent ongoing damage to the heart. Sharma and colleagues showed that Gal-3 was the strongest differentially regulated gene by comparing compensated and decompensated left ventricular hypertension in rats [18, 19]. The assumption that galectin-3 was one of the causes involved in the onset of heart failure was corroborated by infusing Gal-3 into the pericardial sac of wild type rats, which prompted extensive myocardial fibrosis [18, 19]. Other authors had similar results in murine model (angiotensin II-infused mice). Sharma et al. showed that coinfusion of Ac-SDKP along with galectin-3 into the pericardium may play a significant role in cardiac remodeling, not only in inhibiting fibrosis and inflammation but also in alleviating cardiac dysfunction [15].

Fibrosis and scar formation are a part of maladaptive mechanism to the injury. Fibroblasts and myofibroblasts as well as macrophages have been identified as key cells in development and growth of tissue scarring [20, 21]. Upregulation of Gal-3 was found in different human fibrotic conditions (liver cirrhosis, idiopathic lung fibrosis, and chronic pancreatitis) [17, 22–24]. Animal models of hepatic, renal, and cardiac fibrosis have also demonstrated the upregulation of Gal-3 [16, 17, 24]. Galectin-3 mRNA expression was significantly correlated with the extent of fibrosis. Inflammation is essential for tissue healing and scar formation. Sustained inflammation can lead to formation of extensive scar tissue and consequently to organ failure. Macrophages are a key cell type in development of fibrosis [25–27]. Specific depletion of macrophages significantly reduced myofibroblasts activation and decreased fibrosis (characterized by reduced alpha-SMA and collagen expression) [16].

The same authors demonstrated that disruption of Gal-3 gene did not affect macrophage recruitment or macrophage proinflammatory cytokine profiles in response to interferon *γ* [16]. On the other hand, complete macrophage depletion in the Ren-2 rat model accelerated cardiac remodeling, supporting the notion that macrophages have a crucial role in remodelling [28]. We can conclude that galectin-3 and macrophages are major mechanisms in myofibroblast accumulation and activation, as well as in final fibrosis development.

## 3. Galectin-3 and Heart Failure with Reduced Ejection Fraction

The maladaptive changes that occur in surviving myocytes and in the extracellular matrix after myocardial injury lead to pathologic remodelling of the left ventricle, with dilatation and impaired contractility [29]. If these changes are left untreated, they worsen over time, exacerbated by additional injury and by systemic responses to left ventricular systolic dysfunction, notably activation of the sympathetic and renin-angiotensin-aldosterone systems [30, 31]. All these responses have detrimental systemic effects, accounting for the clinical manifestations of the syndrome of heart failure, including the development and worsening of symptoms, declining functional capacity, episodes of frank decompensation that result in the need for hospitalization, myocardial electrical instability, and premature death, usually due to pump failure or a ventricular arrhythmia [29]. Since the limited cardiac reserve of patients with systolic heart failure depends on atrial contraction and synchronized contraction of the left ventricle, events that affect these functions or that impose an additional hemodynamic load on the failing heart can lead to acute deterioration. Interruption of left ventricular remodeling and of the systemic responses to it is the basis of much of the effective treatment of heart failure [29].

The majorities of patients with HF have coronary artery disease and ischemic cardiomyopathy with zones of myocardial fibrosis as a respond to myocardial ischemia and infarction. Dilated cardiomyopathy may also result from a genetic cause, previous viral infection (recognized or unrecognized), alcohol abuse, or, occasionally, chemotherapy [29].

Increased stress and injury of the myocardium can contribute to myocardial remodeling [7]. Respond to myocardial damage involve recruitment of immune cells to the myocardium and production of signaling proteins from local pericytes, mast cells, and macrophages, resulting in activation of resident fibroblast and deposition of procollagen into extracellular matrix which leads to cardiac fibrosis. In the myocardium, aldosterone is a major stimulus for macrophages secretion of galectin-3, which in turn works as a paracrine signal on fibroblast to help translate the signal of transforming growth factor-*β* to increase cyclin D1 and direct the proliferation of myofibroblast and collagen deposition [32]. Galectin-3 is the most upregulated protein in an animal model of left ventricular hypertrophy and HF [15]. Recombinant galectin-3 induced cardiac fibroblast proliferation, collagen production, and cyclin D1 expression. The same investigators demonstrated that intrapericardial infusion of galectin-3 into healthy rats increased left ventricle collagen density and reduced ejection fraction of left ventricle for 22%. These data strongly suggest that galectin-3 is crucial for development of HF [15, 33]. Progressive cardiac fibrosis is central aspect of progressive systolic heart failure leading to creating tissue heterogeneity and stiffness that can cause sudden cardiac death by malignant arrhythmias [29, 32].

## 4. Galectin-3 and Heart Failure with Preserved Ejection Fraction

Diastolic dysfunction or heart failure with preserved ejection fraction (nonsystolic HF) represents an abnormality of diastolic distensibility, filling, or relaxation of the left ventricle, regardless of whether ejection fraction is normal or abnormal and whether the patients are with or without symptoms [30]. HFpEF has a different prognosis and treatment approach than HFrEF. Nonsystolic HF poses a challenge to diagnose with imaging modalities and the set of associated comorbidities such as advanced age, renal disorders, and diabetes [34]. It is known that angiotensin II directly and via stimulation of aldosterone is a crucial neurohormone involved in pathogenesis of cardiac fibrosis and impaired myocardial relaxation [34].

Zile et al. had demonstrated in one small series that galectin-3 levels were significantly elevated in cohort of patients with HFpEF [35]. Galectin-3 might provide an early warning marker for patients who are at risk for development of HF symptoms and may allow medical intervention. According to other animal and human studies, galectin-3 in addition to clinical and echocardiographic parameters can be used to confirm the presence of impaired diastolic function. Some studies have shown that galectin-3 had independent prognostic value, even after correction for established risk factor such as age, sex, BNP level, renal function, and diabetes mellitus [36]. Prognostic value of galectin-3 levels in plasma appears to be much stronger in the subset of patients with HFpEF in comparison with HFrEF [37]. Also, base line levels of galectin-3 seem to be sufficient to predict outcome, because serial measurement did not increase the prognostic yield [36].

## 5. Galectin-3 as Biomarker

Until recently, the goal of HF treatment was based on symptomatic relief. According to new trials and knowledge of myocardial remodelling as a crucial factor in HF development, slowing or reversing the progression of the disease is recognized as important goal of novel therapy (e.g., inhibition of angiotenzin-renin-aldosteron system) [36]. Identifying Gal-3 as an important segment in development of both myocardial remodelling and heart failure has opened possibility of Gal-3 being used as a new marker for the disease prognosis as well as a new treatment target.

Brain natriuretic peptide (BNP) and N-terminal pro-brain natriuretic peptide (NT-proBNP) assay were a golden standard in prognosis of HF in the past years. Natriuretic peptides are relisted by the myocardium as a result of myocardial stretching. In normal condition their level vary widley. In heart diseases condition, it is a marker of worsening heart failure and can present practical tool for heart failure treatment [37]. Troponin T and Troponin I (TnI/TnT) assay is useful in HF patients and according to trial data, slight elevations or chronically elevated levels of TnI/TnT predict a poor outcome [38]. Raised TnI/TnT levels are highly specific for myocyte injury. They are primarily used as markers in acute myocardial infarction, as well as in heart failure and some other conditions [39]. Like NTproBNP/BNP, the circulating TnI/TnT level is not pathogenic and specific and can be viewed as a signal of an ongoing pathological process in the heart. On the other hand, Gal-3 complements other HF biomarkers by providing an upstream signal of the myocardial fibrotic state, ventricular adverse remodelling, and progression of cardiomyopathy. Considering that cardiac fibrosis is irreversible process, Gal-3 measurement provides serological overview of the ongoing fibrotic process. BNP/NT-proBNP, TnT/TnI, and Gal-3 aid in prognosis, risk stratification, and management. Gal-3 levels are a direct reflection of cardiac fibrosis, are not acutely changed by HF decompensation, stay elevated once elevated in majority of cases, and are not affected by medical treatment [40]. DEAL-HF trial showed that plasma galectin-3 level has a prognostic value regardless of heart failure severity, as assessed by NT-proBNP levels, and it may be potentially used in management of such patients [41]. Galectin-3 and its prognostic value have been evaluated in number of studies. In 240 patients with stable chronic HF plasma Gal-3 levels were strongly related to outcome [42]. In another trial, data for 599 patients presented with dyspnoea at the emergency department were analyzed by receiver operating characteristic analysis. The results showed that in two-month period mortality was higher in patients with higher plasma galectin-3 level, presenting with a greater area under the curve at 0.74 compared with NT-proBNP [37]. Multivariate logistic regression analysis revealed that elevated plasma galectin-3 level was the best independent predictor of 60-day mortality or combination of death/recurrent HF within 60 days. Milting et al. found significantly elevated plasma galectin-3 levels at the time of mechanical circulatory support [43]. Patients who died had significantly higher plasma Gal-3 level than those who were transplanted. This is also one more argument that galectin-3 plays an important role in myocardial remodelling and HF development. Galectin-3 is also associated with the risk of developing HF after acute coronary syndrome and supports potential clinical relevance of galectin-3-related pathway in patients with ischemic heart disease [44]. Galectin-3 was a strong independent predictor of 30-day major adverse cardiac outcome among patients with STEMI infarction undergoing primary PCI and thus can be utilized as a useful biomarker for stratifying high and low risk subgroups in daily clinical practice [45].

As mentioned before, various fibrotic conditions are associated with upregulation of galectin-3. Not only heart fibrosis but also upregulation of galectin-3 has been described in animal model for hepatic and renal fibrosis; in human liver cirrhosis; and in idiopathic lung fibrosis and chronic pancreatitis [16, 17, 46–48]. When taking into consideration importance of galectin-3 in heart failure pathophysiology, certainly we have to exclude the impact of these conditions on the final conclusions. Despite the fact that the frequent companion of heart failure is cardiorenal syndrome (renal failure), after correction for established risk factor (diabetes, age, sex, and renal function) it has been proven that high levels of galectin-3 has independent and significant impact on the prognosis of patients with heart failure [36, 49].

## 6. Conclusion

In every day clinical practice Gal-3 can be used to identify those patients at highest risk for readmission or death of HF. Galectin-3 measurement may be a significant factor in making a decision regarding visit intervals of whom to admit in patients with worsening HF. Due to the fact that Gal-3 levels are directly correlated with remodelling and fibrotic process in the myocardium, Gal-3 can be used as a culprit biomarker and can contribute to heart failure treatment as a potential novel target in therapeutics. This would be a real disease-modifying therapy to inhibit the remodelling process or slow down HF progression. Galectin-3 may one day allow identification and treatment of patients with coronary artery disease with a major risk of cardiomyopathy development.
